# Immunochemical detection of mycotoxins in donkey milk

**DOI:** 10.1007/s12550-018-0333-2

**Published:** 2018-10-20

**Authors:** Madeleine Gross, Christian Puck Ploetz, Christoph Gottschalk

**Affiliations:** 1 Faculty of Veterinary Medicine, Institute of Veterinary Food Science, Junior Professorship of Veterinary Food Diagnostics, Justus-Liebig-University, Ludwigstr. 21, 35390 Giessen, Germany; 2 Chair of Food Safety, Faculty of Veterinary Medicine, Ludwig-Maximilians-University Munich (LMU), Schoenleutnerstr. 8, 85764 Oberschleissheim, Germany

**Keywords:** Donkey milk, Mycotoxin, Aflatoxin, Ochratoxin A, Zearalenone, Enzyme immunoassay, Lysozyme

## Abstract

The applicability of enzyme immunoassays (EIA) for aflatoxin M_1_ (AFM_1_), ochratoxin A (OTA) and zearalenone (ZEN) to analyse these toxins in donkey milk (*Equus asinus*) was studied. For AFM_1_ and OTA analysis, milk could be analysed by EIA without sample pretreatment. For ZEN, heat treatment at 78 °C for 30 min prior EIA analysis was required to avoid false positives. To include detection of phase II metabolites of ZEN, samples were additionally treated with glucuronidase/sulfatase for this EIA. Detection limits were 5 ng/kg (AFM_1_), 9 ng/kg (OTA) and 600 ng/kg (ZEN). All donkey milk samples were negative for all three toxins. Satisfactory quantitation was achieved for spiked samples. Analysis of some cereal-containing donkey feed components (pellets, oats) by EIA revealed absence of aflatoxin B_1_ (AFB_1_, < 3 μg/kg) and OTA (< 4 μg/kg), while ZEN was found in pellets (180 μg/kg) and in oats (7 μg/kg). This is the first one study on multitoxin determination in donkey milk by antibody-based test systems. In general, the results confirm that EIAs are convenient tools for mycotoxin detection in donkey milk. However, false-positive results may occur, possibly due to the high lysozyme content of donkey milk, which may exert inhibitory activity in some competitive EIA systems. Therefore, specific validation of each EIA for this specific matrix is required, and re-analysis after heat treatment of EIA-positive donkey milk is highly recommended.

## Introduction

The total world population of donkeys (*Equus asinus*) is about 43 million; the vast majority (98%) lives in Africa, Asia and middle/south America (FAOSTAT 2018). In Europe, agricultural use of donkeys for meat and milk production is of minor economic importance. However, in the Mediterranean and in the Balkan areas, donkey meat, donkey milk (powder) and donkey milk cheese (pule) are produced at a small scale. Recently, research interest in donkey milk has increased in Europe, especially in Italy (Camillo et al. 2018). Donkey milk is claimed to be similar to human breast milk in nutrient composition (Silanikove et al. 2016), although analytical data proved otherwise (Guo et al. 2006; Alichanidis et al. 2016; Aspri et al. 2016). Further, it is an alleged alternative in cases of cows’ milk intolerance (Monti et al. 2012; Polidori et al. 2009; Carrocino et al. 2000), but lysozyme which is abundant in donkeys milk recently was identified as a major allergen (Martini et al. 2018).

Donkey milk distinctly differs in composition from cows’ milk or human milk, with very low fat (0.1–1.8%) and protein (1.5–1.8%) contents but a high lactose (5.8–7.4%) content (Guo et al. 2006; Alichanidis et al. 2016). Like mare’s milk, and in contrast to milk from ruminants, donkey milk has a very high content of an inhibitory enzyme, lysozyme, with maximum reported levels up to 1.4 g/l (Salimei et al. 2004) or even 4 g/l (Brumini et al. 2016).

As a monogastric species, the digestive system of equines strongly differs from that of ruminants. Consequently, data obtained from cows concerning mycotoxin carry over into milk are not per se applicable to donkeys. Donkey feed typically consists simply of grass, hay and minerals. In case of professional donkey breeding farms, and for lactating animals, cereals and cereal-based pellets are added to the diet (Aganga et al. 2000). Cereals are a relevant source for mycotoxin intake and consequently might result in a carry-over into milk. In the European Union, maximum levels for mycotoxins in animal feed have only been set for AFB_1_, at 5 μg/kg (European Communities 2002). For deoxynivalenol, ZEN, OTA, fumonisins (FB_1_/FB_2_) and T2/HT2 toxins, the European Commission has published guidance values in various feeds and for several livestock species (European Commission 2006a). Except for fumonisins (5 mg/kg in feed), none of these recommendations specifically is dedicated to equine feeding stuff, and donkeys are not mentioned in this document at all. As a non-ruminant monogastric species, donkeys may be more sensitive towards adverse effects of mycotoxins, but again, little is known here except a specific sensitivity towards fumonisins, the causal agents for fatal leukoencephalomalacia in donkeys (Rosiles et al. 1998; Gross et al. 2015).

Published studies on carry-over from feed and natural occurrence of mycotoxins in donkey milk are scarce and, to our knowledge, most exclusively deal with aflatoxins (Escrivá et al. 2017). Tozzi et al. (2016) fed a diet containing 1 kg maize per day which was contaminated with aflatoxin B_1_ (AFB_1_, 202 μg/kg) and aflatoxin B_2_ (AFB_2_, 11 μg/kg) to six Romagnola breed donkeys for 12 days. They found low levels of 32 ng/l of AFM_1_ and up to 23 ng/l of AFM_2_ in the milk of all animals, and calculated carry-over rates of 0.2% for AFM_1_ and 0.31% for AFM_2_. This is much lower than the 2–6% carry-over rates reported for cows (Britzi et al. 2013). In a study on 90 donkey milk samples from seven farms in Greece and Cyprus, Malissiova et al. (2016) did not detect any AFM_1_ positives with a commercial EIA test kit. More recently, using a commercial AFM_1_ EIA test kit from another producer, Malissiova and Manouras (2017) obtained AFM_1_-positive results (5–26.5 ng/l) for 5 out of 36 donkey milk samples from Greece farms. Considering the results of Tozzi et al. (2016), some feed compound ingested by these animals must have been contaminated with AFB_1_ at very high levels to achieve such results in milk, but unfortunately, Malissiova and Manouras (2017) did not analyse relevant feed components for AFB_1_, or provided an explanation for their findings. Using yet another commercial EIA for AFM_1_, Bilandzic et al. (2014) obtained AFM_1_-positive results of 2–10 ng/l in donkey milk from Croatia (14 samples), but did not report the number of positives. An AFM_1_ EIA test kit from a fourth producer was used to analyse milk from donkeys in Serbia, and 3 out of 5 samples were AFM_1_ positive, at levels of 3–35 ng/kg (Kos et al. 2014). Although neither Bilandzic et al. (2014) nor Kos et al. (2014) included feed analyses in their studies, these two surveys were possibly influenced by high AFB_1_ contamination of maize in some Balkan countries in 2012 (Kos et al. 2013) which subsequently lead to elevated AFM_1_ levels in cows’ milk. This is supported by a recent study (Cammilleri et al. 2018) which found no AFM_1_ in 84 samples of donkey milk from Sicily, Italy, during 2013–2016.

For ZEN, only one study (Capriotti et al. 2015) analysed donkey milk by liquid chromatography-mass spectrometry (UHPLC-ESI-MS/MS), but all five samples were negative at a claimed detection limit of 20 ng/kg.

In this situation, a more specific assessment of the situation with regard to mycotoxins in donkey milk seems to be required. Commercial mycotoxin EIA test kits for mycotoxins in milk are available for only for AFM_1_. These have been validated for cows’ milk, in some cases for sheep and goats’ milk, but not for equine milk. However, it is questionable if validation data for cows’ milk are applicable to donkey milk because it is a strongly differing matrix. For other mycotoxins, there is a complete absence of EIA application studies using donkey milk. Therefore, the aim of this study was to assess the applicability of three mycotoxin EIAs for AFM_1_, OTA and ZEN for analysis of donkey milk, as part of a project to establish simple and versatile screening systems for residues and contaminants in equine milk.

## Materials and methods

### Sample materials

Samples were obtained from a small herd of Baudet du Poitou donkeys in Germany. In this herd, six mares were lactating. Each one milk sample (about 50 ml) of the six lactating donkeys, which had been collected during routine herd management, was available for analysis. The average age of these animals was 7.5 ± 2.7 years; the average body weight was 350 kg. During this study, the feed of the animals consisted of grass and hay ad libitum. Additionally, a single batch of each a commercial pelleted feed (containing oat fibre, wheat bran and maize), purchased from a local distributer and oat grains from a non-commercial source in the area of Giessen, Germany, were fed to these animals. The amount of these feeds consumed per animal per day was not controlled, but was estimated 0.2–0.3 kg of pellets and 0.5 kg oats per day. While grass and hay were not analysed for mycotoxins, a 3-kg sample of each pellets and oats was collected. This sample was manually mixed in a 10-l plastic bucket, and a subsample of 500 g was collected. These samples were ground to a mean particle size of < 1 mm in a laboratory mill, and manually mixed again before each series of analyses. These samples were analysed for OTA and ZEN by competitive direct EIA as described previously (Liesener et al. 2010; Gross et al. 2015), while the EIA for AFB_1_ (Gathumbi et al. 2001) was used with a sample preparation method described by Märtlbauer et al. (1991). Neither AFB_1_ (detection limit 3 μg/kg) nor OTA (detection limit 4 μg/kg) was found in pellets and in oats, the ZEN-EIA determined 180 μg/kg in pellets and 7 μg/kg in oats.

## EIA analysis

Polyclonal rabbit antisera and toxin-horseradish peroxidase conjugates for competitive direct EIAs for ZEN and OTA were used as described previously (Usleber et al. 1992; Schneider et al. 2001). For AFM_1_, a commercial enzyme immunoassay test kit (R-Biopharm, Darmstadt, Germany, R1111) was used according to manufacturer’s instructions, except that milk samples were not defatted before analysis. For analysis of AFM_1_ and OTA, 50 μl of milk sample per well were directly analysed by EIA without any further purification or dilution.

For ZEN-EIA analysis, preliminary studies had indicated the likelihood of false-positive results when using raw milk samples. Therefore, milk samples were heated at 78 °C in a water bath for 30 min, and then cooled to room temperature for analysis of ZEN. Also, exclusively for the analysis of ZEN, a 2 ml portion of milk was mixed with 24 μl of ß-glucuronidase/sulfatase (Sigma Aldrich, G7017, approx. 2200 U/ml milk), incubated for 2 h at 37 °C, and then diluted 1:6 with EIA buffer (5% acetonitrile/PBS pH 7.2). Fifty microlitre per well were then analysed by the ZEN-EIA.

The EIA standard curve cutoff values (detection limit, LOD) for OTA and ZEN were set at a toxin concentration resulting in 20% binding inhibition compared to the negative control (IC_20_), corresponding to the end of the semilinear range of the standard curve. Four replicates of all standard and sample solutions were analysed, and each milk sample was analysed in at least two different dilutions, to check for sample matrix effects. For the AFM_1_ EIA, the detection limit was equal to the standard with the lowest toxin concentration (5 ng/kg). For this test, milk samples were analysed in duplicate wells only.

To study sample matrix interferences, milk samples were artificially contaminated with different concentrations of toxin standard solutions before analysis.

## Results and discussion

All three EIAs enabled sensitive and simple detection of the respective mycotoxin analysed. With regard to AFM_1_, the detection limit of 5 ng/kg was five times lower than the lowest maximum level (25 ng/kg) set by European Union regulation 1881/2006 in milk-based food for infants (European Commission 2006b). For OTA, no specific regulation according to European Union regulation 1881/2006 for milk exists, but the lowest maximum level set for “dietary foods for special medical purposes intended specifically for infants” is at 500 ng/kg. The detection limit of the OTA EIA (9 ng/kg) clearly was below that. For ZEN, which is not regarded as a relevant contaminant in cows’ milk (Kappenstein et al. 2005), the lowest maximum level according to European Union regulation 1881/2006 of 20 μg/kg is set for “processed cereal-based foods (excluding processed maize-based foods) and baby foods for infants and young children”. Again, the ZEN-EIA enabled detection of this toxin in donkey milk well below this level, with a detection limit of 600 ng/kg.

**Fig. 1:**
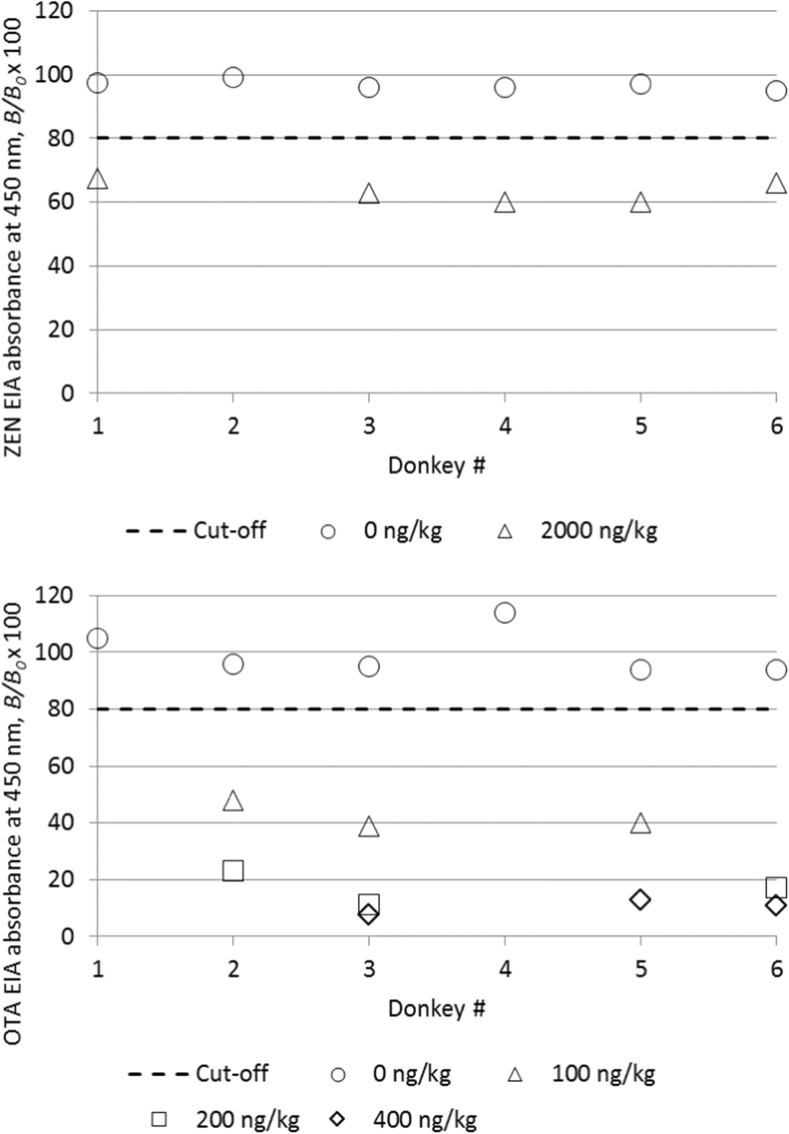
Comparison of EIA absorbance readings for blank and corresponding spiked milk samples from six donkeys in the EIAs for ZEN and for OTA. Spiking level was 2000 ng/kg for ZEN, while for OTA, three different spiking levels (100, 200 and 400 ng/kg) were tested. Not all samples were spiked with each concentration of toxin

All six samples were clearly negative for AFM_1_ and OTA, which is plausible considering the negative results for AFB_1_ and OTA in pellets and oats, which were part of the donkeys’ diet, and assuming that grass and hay are not major sources of intake for toxins. For the AFM_1_-EIA, no further validation was done within this study, but recovery of AFM_1_ from mares’ milk at levels of 5–15 ng/kg had been found at 91–133% (unpublished results). In the OTA-EIA, donkey milk yielded absorbance values around the standard curve blank (*B*_0_), as shown in Fig. 1. Donkey milk spiked at 100, 200 and 400 pg/kg yielded toxin results of 44–77% of the nominal values, with coefficients of variation ranging from 4.3–20%. Currently, no information is available on the risk of OTA contamination in donkey milk, but it has been found in blood of 83% of horses at levels up to 700 pg/ml (Minervini et al. 2013), which suggests that transfer into milk of lactating donkeys is possible depending on the intake of this toxin. The OTA-EIA could be a convenient tool to screen donkey milk samples.

For the ZEN-EIA, preliminary experiments had shown that all six-donkey milk samples resulted in binding inhibition of about 30–40% (60–70% *B*/*B*_0_), even for diluted samples. Since such results seemed to lack plausibility in the light of low to moderate ZEN levels in pellets and oats, milk sample matrix interference was considered as a possible cause. Although the ZEN-EIA has successfully been used and validated for the analysis of ZEN in raw cows’ milk (Usleber et al. 1992), the high levels of lysozyme, which is a protein with antimicrobial properties, in donkey milk were assumed to be a possible cause of the observed inhibition. Lysozyme is practically absent in the milk of cows, sheep and goats, but levels up to 4 g/l have been found in donkey milk (Brumini et al. 2016). Lysozyme is quite heat stable, and 62 °C for 30 min or 90 °C for 1 min did not eliminate it from milk (Coppola et al. 2002), temperatures higher than 60 °C may even increase activity due to dissociation of di- and trimers (Vincenzetti et al. 2017). In contrast, Ozturkoglu-Budak (2018) reported a 60% decrease of lysozyme at 85 °C for 2 min. Although no explanation of the possible mode of action of lysozyme on enzyme immunoassay could be provided here, heating donkey milk at 78 °C for 30 min strongly weakened the competitive binding inhibition in the ZEN-EIA and increased absorbance readings to about 80–85% *B*/*B*_0_. Such sample matrix effects seemed to be test specific, since they were not observed in the AFM_1_ EIA and the OTA EIA. Therefore, it could be due to effects on antibody binding rather than on toxin-horseradish peroxidase activity, but further studies are required on this phenomenon. Dilution of heat-treated samples 1:6 with buffer solution yielded absorbance values close to the standard curve blank value (*B*_0_), as shown in Fig. 1. Results did not change with or without addition of glucuronidase/sulfatase, indicating that phase II metabolites of ZEN were not present at relevant levels in these samples. Artificially, contaminated milk samples at a level of 2000 pg/kg were clearly positive (Fig. 1), resulting in ZEN-EIA results of 62 ± 7% of the nominal value. At present, it is unknown whether or not ZEN could be an issue in donkey milk. In comparison with a standard curve detection limit of some improvement of the sensitivity in milk seems to be desirable for this assay. The standard curve detection limit of 100 ng/kg provides sufficient sensitivity to achieve some further improvement.

The observations made on the putative lysozyme–related matrix effects on the ZEN-EIA should be considered for the evaluation of previously reported results for AFM_1_ in donkey milk. Based on the results of Tozzi et al. (2016) who showed that carry-over of AFB_1_ as AFM_1_ into donkey milk is minimal compared with cows’ milk, positive AFM_1_ results in milk require very high contamination levels of feed compounds which are fed in addition to grass and hay. Results as reported by Malissova and Manouras (2017) or by Kos et al. (2014) require very high AFB_1_ levels in feed exceeding 100 μg/kg, a level which hardly could be unobserved for prolonged periods, also in the light of European Union regulations (5 μg/kg) for AFB_1_ in feed for lactating animals (European Communities 2002). Assuming that lysozyme could also negatively affect commercial competitive mycotoxin EIAs in a similar way as the ZEN-EIA, control analyses are advisable for such tests. Reanalysis of positive samples after heat treatment, for example, 78 °C for 30 min as used in this study, would be one option. Further, positive results in donkey milk are certainly not a routine occurrence in food hygiene but demand mycotoxin analysis in feed, to identify possible sources of the toxin. A third option would be the confirmation of positive results by independent methods, which may be more difficult in case of AFM_1_ than for OTA or ZEN. In any case, EIA validation data obtained for cows’, sheep or goats’ milk are of limited value for donkey milk analysis. Control tests, including analysis of confirmed negative samples and spiked negative samples, and checking for effects of heat treatment, are essential when donkey milk should be analysed for mycotoxins by enzyme immunoassay techniques.
